# Complementary food exposure and children’s early understanding of food words: the approaching eating through language (APPEAL) study

**DOI:** 10.3389/fnut.2024.1237698

**Published:** 2024-05-28

**Authors:** Allison L. B. Shapiro, Megan C. Lawless, Renee Charlifue-Smith, Susan L. Johnson

**Affiliations:** ^1^Section of Endocrinology, Department of Pediatrics, University of Colorado Anschutz Medical Campus, Aurora, CO, United States; ^2^Lifecourse Epidemiology of Adiposity and Diabetes Center, University of Colorado Anschutz Medical Campus, Aurora, CO, United States; ^3^Section of Nutrition, Department of Pediatrics, University of Colorado Anschutz Medical Campus, Aurora, CO, United States; ^4^JFK Partners, University of Colorado Anschutz Medical Campus, Aurora, CO, United States

**Keywords:** complementary feeding, language acquisition, infants, toddlers, latent class analyses (LCAs)

## Abstract

**Introduction:**

Language skills, such as the ability to understand words (receptive language), develop during infancy and are built through interactions with the environment, including eating. Exposure to complementary foods also begins in infancy and may play a significant role in language development, especially in understanding of food-related words. However, the relationship between the complementary foods to which a child is exposed and early language acquisition has not been previously studied. We hypothesized that young children’s food-related receptive language (FRL) would reflect the complementary foods to which they were frequently offered by caregivers.

**Methods:**

Caregivers of young children (4-26 months; *n* = 408) in the Approaching Eating through Language (APPEAL) Study in the US were surveyed via Qualtrics. FRL was assessed by caregiver-report via a modified MacArthur-Bates Communicative Development Inventory. Complementary foods offered (CFO) by caregivers were assessed using a modified Food Frequency Questionnaire. Latent Class Analysis (LCA) was implemented to identify, 1) groupings of foods frequently offered (>1x/week) and 2) groupings of food-related words understood by the young children.

**Results:**

A 5-class best fit LCA model was identified for CFO (-log likelihood [-llik]=-8727) and for FRL (-llik=-5476). Cross-classification of the CFO and FRL derived classes revealed that children with higher exposure to complementary foods were perceived by caregivers to be most likely to also understand a greater number of food-related words (Probability=0.48). As expected, children having been offered a greater number of complementary foods and who understood a greater number of food-related words were older, compared to those with less complementary food exposure and food-related language acquisition (*p* < 0.001).

**Discussion:**

These findings support the potential role of introduction to complementary foods in development of food-related language.

## Introduction

Complementary feeding in infancy is a critical nutrition and behavioral transition from breast or formula milk feeding to exposure to novel foods and foods of the family. Complementary feeding provides an important addition to the nutrition obtained from breast or formula milk during the weaning phase, as well as influences development of eating preferences and behaviors (1, 2). Importantly, the timing of complementary feeding (4–6 months of age) coincides with other critical developmental processes like language acquisition, motor control, and cognitive function, forming a multidimensional and interactive landscape of learning in the first few years of life.

Language acquisition, in particular, is a cornerstone of the early learning landscape, and acts as a significant driver of a child’s ability to interact with their surrounding environment and strongly influences behavior (3–5). So, too, do the environment and behavior influence language acquisition where, by the tenets of the interactionist framework of language, early development of language is driven by a child’s interaction with their environment and repetition and reinforcement by caregivers (6). Importantly, in early life, feeding and eating are ubiquitous activities and provide significant opportunity for repetition and reinforcement of language that is food-related. Thus, following the interactionist theory of language, feeding and eating may act as significant drivers of a child’s early lexicon, or acquired vocabulary, both understood and spoken.

Children’s’ vocabulary serves as one part of the scaffolding for higher-order cognitive skills (7). For example, language development in preschoolers has been found to longitudinally predict performance on executive function tasks (8, 9). It has also been shown that children with greater task-related vocabulary are better able to understand what they are being asked to do to complete a task in question, thereby improving their performance on said task (10). Similar links may occur within the eating domain, such that greater understanding of or ability to verbalize the words that correspond to foods being offered could influence what a young child is willing to eat. Indeed, evidence from our group supports that toddlers with larger food-related receptive language lexicons were more likely to accept a novel food, compared to toddlers and infants whose food-related vocabulary development was less advanced (11). Thus, we posit that, as children acquire greater food-related language through exposure to foods, they likely build a larger food and eating-based lexical and cognitive network. This, in turn, may contribute to a greater capacity to engage with new foods and food-related experiences. Ultimately, this link may help to shape eating behaviors in early life and thereby influence long-term health outcomes related to food acceptance and dietary patterns as well as picky eating, eating disinhibition, and restrictive eating.

To date, however, few studies have investigated the relationships among early food-related experiences, like the variety of exposure to foods during the complementary feeding period, and food-related language acquisition. Therefore, it is currently unknown whether the food-related words understood by a young child are reflected in the complementary foods they have been offered during this critical developmental window. To begin to address this gap in our knowledge, and to explore children’s development of eating within the context of language learning and the interactionist framework of language development, we undertook a hypothesis generating investigation of the relationship between the complementary foods to which a young child is exposed and early language acquisition of food-related vocabulary.

## Methods

### Participants

The Approaching Eating through Language (APPEAL) Study recruited caregivers of young children who lived in the United States using the online Qualtrics platform (Qualtrics, Provo, UT). Inclusion in the APPEAL Study required that caregivers responding to the questionnaire (“caregiver respondent”) to be English-speaking and speak English in the home, the primary individual responsible for feeding their child (feeds child >50% of time), ≥18 years and <51 years of age, and the primary caregiver of a young child between the ages of 4- and 26-months old (“index child”). English-speaking was required due to the language assessment being administered (see Methods). Further, the index child must have already begun introduction to solid/complementary foods (i.e., rice cereal or other solid food) prior to participation. Caregivers were excluded if the index child was reported to have a genetic disorder or developmental disability or was born prematurely (<37 weeks’ gestation), as these conditions often result in feeding difficulties. Caregiver respondents were further excluded if they reported that the index child had a language delay or food allergies of any kind.

**Figure 1:**
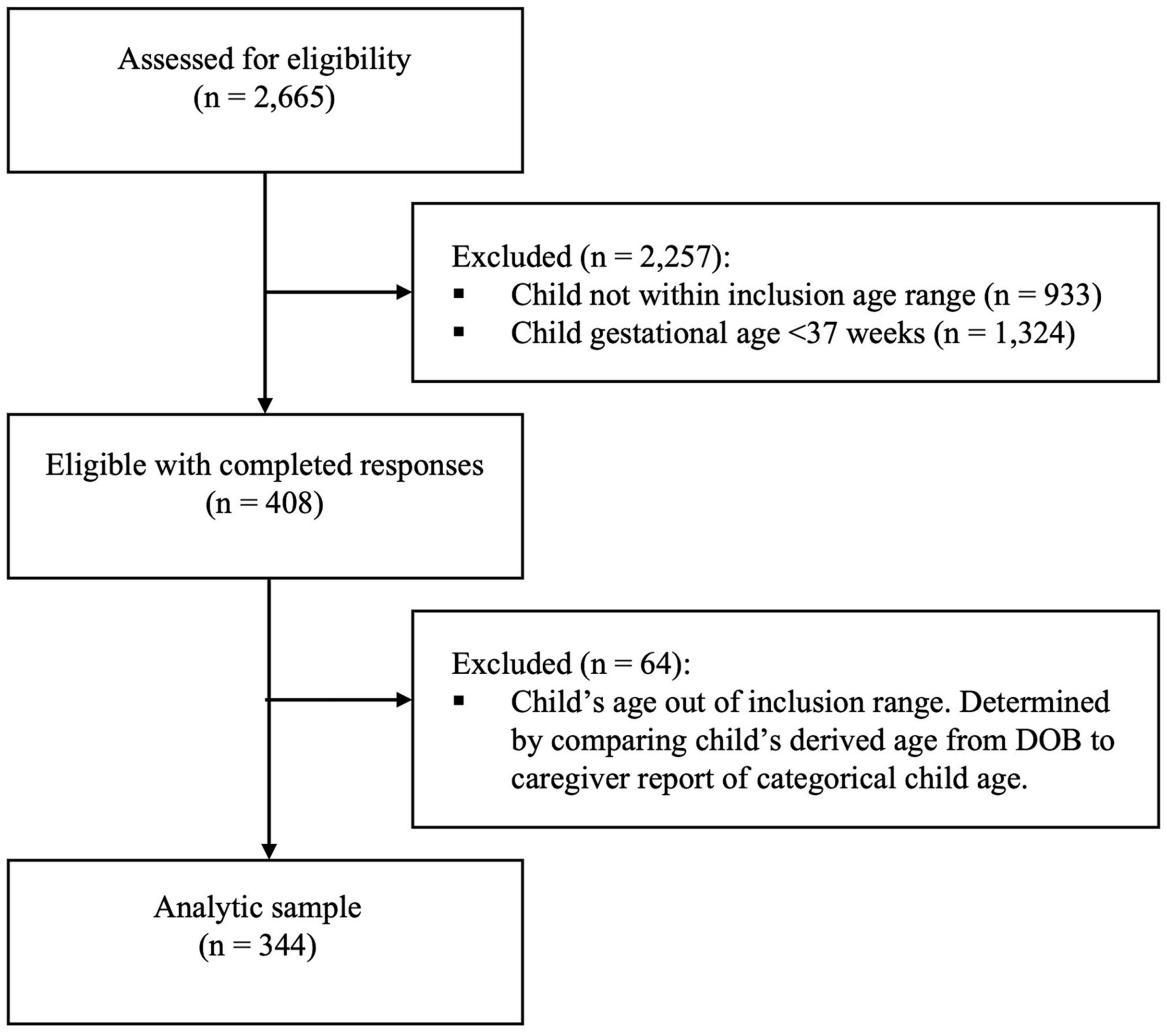
Sample size flow chart.

Methods used to distribute the survey did not allow for determination of the number of people who viewed the invitation. A total of 2,665 caregivers met inclusion criteria and from whom survey responses were started in Qualtrics (opened 11/5/21) before data collection was closed on 12/10/21. Of these caregivers, Qualtrics removed a substantial number of responses that were incomplete, from duplicate participants, or poor-quality responses (e.g., straight-lined responses). This resulted in a final sample size of n = 408 caregiver respondents. A further 64 caregiver respondents were excluded due to: (1) inconsistencies in the reported categorical age of the index child and their calculated age, which was derived as the difference between their date of birth and date of survey completion; or (2) the calculated age of the index child was outside of the eligibility age range. These additional exclusions resulted in an analytic sample size of 344 caregiver-child dyads (Figure 1).

### Questionnaire development

We implemented questionnaire development methods proposed by Dillman (12) for internet-based designs. Our questionnaire presented questions in blocks of in-house developed items or items derived from validated questionnaires. The question blocks included a screener block (e.g., child age, caregiver age, food allergies, etc.; 10 items); caregiver and child demographics block (e.g., household income, race and ethnicity, etc.; 18 items); child demographics block, infant feeding history block (e.g., child ever breastfed, age when breastfeeding stopped, etc.; 5 items); a modified Block Food Frequency Questionnaire (13) (see below for further description; 57 items); a modified MacArthur Bates Communicative Development Inventory (see below for further description; 73 items), among several other questionnaire blocks. A full copy of the measures and items included in the APPEAL Study questionnaire can be found on the Open Science Framework site at: https://doi.org/10.17605/OSF.IO/TA56P.

Before release of our questionnaire to Qualtrics, it was tested via cognitive interviewing performed on a sample of 3 female caregivers, 18–51 years-old, whose children were between the ages of 6 and 24 months old. Upon release of our survey to Qualtrics, we defined a sampling frame for race and ethnicity (Non-Hispanic White [66%], Non-Hispanic Black [12%], Hispanic [12%], Other or multiracial [10%]), specifically oversampling for people of color.

### Caregiver and child sociodemographic information

Caregiver respondents were asked to report their biological sex (female, male, and intersex), race (White, Black or African American, American Indian or Alaska Native [AI/AN], Asian, Native Hawaiian or Pacific Islander [NW/PI], or a race not listed) and ethnicity (Hispanic, Latinx, Chicano or of Spanish origin). In the current analysis, race and ethnicity were collapsed into three categories due to low cell counts: non-Hispanic white (NHW); non-Hispanic black (NHB); Hispanic, or other, or more than one race and ethnicity. Caregiver respondents also reported their age (years), household income (<$26,500; $26,500–$50,000; $50,000–$100,000; and >$100,000), number of adults living in the household, number of children living in the household, and their highest level of educational attainment (some high school, high school or GED, some college, 2-year degree, 4-year degree, post-grad or professional degree, graduate degree). Caregiver respondent education was collapsed into two groups: high school degree or less, more than high school degree.

Caregiver respondents reported on their relationship to the index child (mother, father, grandmother, legal guardian, grandfather, and other), the index child’s biological sex (female, male, and intersex), date of birth, and birthweight (pounds [lbs] and ounces [oz]). Child age was collapsed into <12 months, 12–18 months, and >18 months for descriptive purposes.

### Food-related language

The primary outcome measure for this analysis was caregivers’ perceptions of their children’s food-related vocabulary, as measured by a modified version of the MacArthur Bates Communicative Development Inventory (MB-CDI). The MacArthur Bates Communicative Development Inventory (14) is a caregiver self-report catalog of words that reflects the caregiver’s perception of their infant/toddler’s ability to understand words (receptive language) and speak words (expressive language). For the APPEAL Study, we modified the language inventory to include only words that were food words (e.g., apple) or food-related words, like eating utensils (e.g., fork). As the MB-CDI has been validated and used widely in language screening, and in an effort retain the structure from the validated instrument, we chose to include all food words and food item words verbatim from the MB-CDI. The food words included from the MB-CDI were cross-referenced with the most commonly consumed complementary foods reported by the Feeding Infants and Toddlers Study (15, 16). Indeed, we found significant overlap, thus, confirming that the food words included in our modified MB-CDI were representative of common foods offered to infants and toddlers. A full list of words can be found in Supplementary Table S1.

Response options for each word item included, “cannot understand or say,” “can understand,” and “can understand and say.” The caregiver respondents were instructed to select a single response that best described their perception of the index child’s language ability. In the current analysis, we investigated receptive language only and only those words from the MB-CDI that were food words, excluding food-related words (e.g., refrigerator).

### Complementary foods offered

Complementary foods are defined as any food other than infant formula or breast milk. In the APPEAL Study, introduction of complementary foods was assessed using a modified Block Food Frequency Questionnaire (13) (mFFQ) that recorded frequency of offering the 57 foods that were listed on the MB-CDI. Included in the list of 57 foods were 32 “adequacy” foods, or foods that contribute to meeting the recommended nutritional needs for children 2 years old and younger, and 25 “moderation” foods, or foods that are energy dense but nutritionally poor or are recommended to be consumed infrequently or not at all (17). Response options for frequency of offering each food within the previous 30 days were “not at all,” “a few times,” “once a week,” “a few times per week,” “daily or more than once per day.” For the current analysis, frequency categories were collapsed into “not at all or a few times” vs. “at least once per week” to capture consistency of the complementary foods being offered.

### Statistical analyses

Given the large list of foods and food words queried in the APPEAL Study, we chose to investigate patterns of adequacy and moderation of the complementary foods offered by caregivers and patterns of understanding of adequacy and moderation food words, and the relationship of these patterns to each other. We implemented a data-driven clustering method, specifically latent class analysis (LCA) (18) via the R package PoLCA. LCA seeks to maximize the between-cluster differences and minimize the within-cluster differences among individuals in a sample. We tested several iterations of the LCA models where we *a priori* specified different expected numbers of classes, ranging from 3 to 6. We compared LCA models for best fit using standard model fit statistics including the negative log likelihood (model with lowest negative value is best fit) and the Bayesian Information Criterion (BIC; model with lowest BIC value is best fit) (19).

In our first modeling step, we applied LCA to the set of variables for complementary foods offered (CFO) to achieve data reduction, which resulted in a 5-class best fit model [CFO LCA; log-likelihood(5) = −8726.648, BIC(5)=19141.24]. In our final modeling step, we ran a latent class regression model, whereby the derived CFO classes were included as the main predictor variable and the set of food words (FW) from the MB-CDI were set as the outcomes. Here, we tested how well CFO classes predicted FW class membership, and estimated the probability of each of the FW classes [FW LCA 5-class best-fit model; log-likelihood(5) = −5475.706 BIC(5) = 12662.72] belonging to each of the CFO classes. In other words, we examined how the complementary food items that were offered and that clustered together predicted the food words that were understood and that also clustered together.

**Figure 2:**
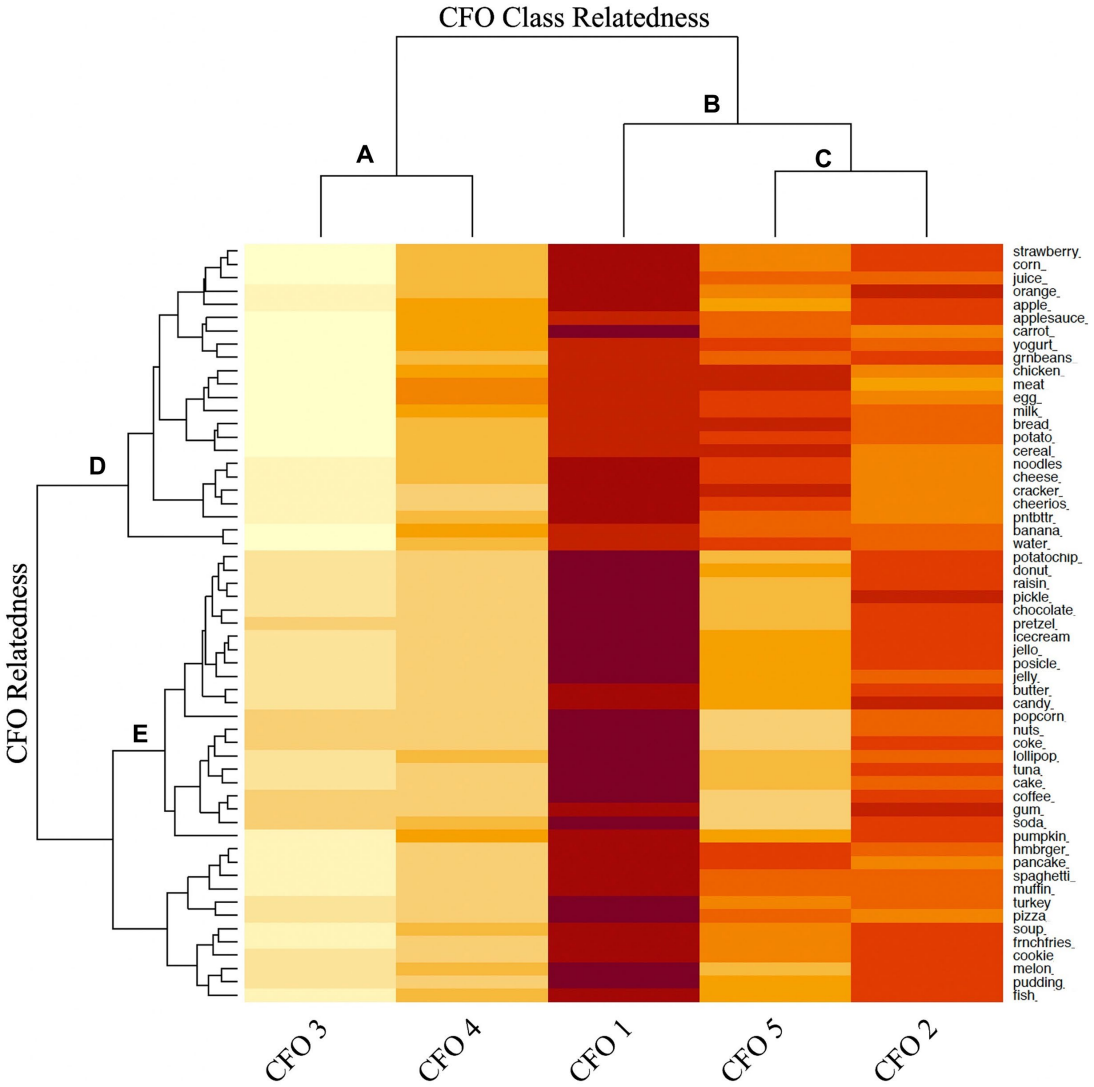
Latent class analysis of complementary foods offered (CFO) heat map. In order of increasing complementary food exposure: CFO Class 3 = low exposure to adequacy and moderation complementary foods; CFO Class 4 = low-to-moderate exposure to adequacy and moderation complementary foods; Class 5: moderate exposure to adequacy and moderation complementary foods; CFO Class 1 = high exposure to adequacy and moderation complementary foods. The dendrogram displays the similarity or relatedness of the derived classes **(A–C)** and the similarity of individual complementary food items understood in each class **(D,E)**.

**Figure 3:**
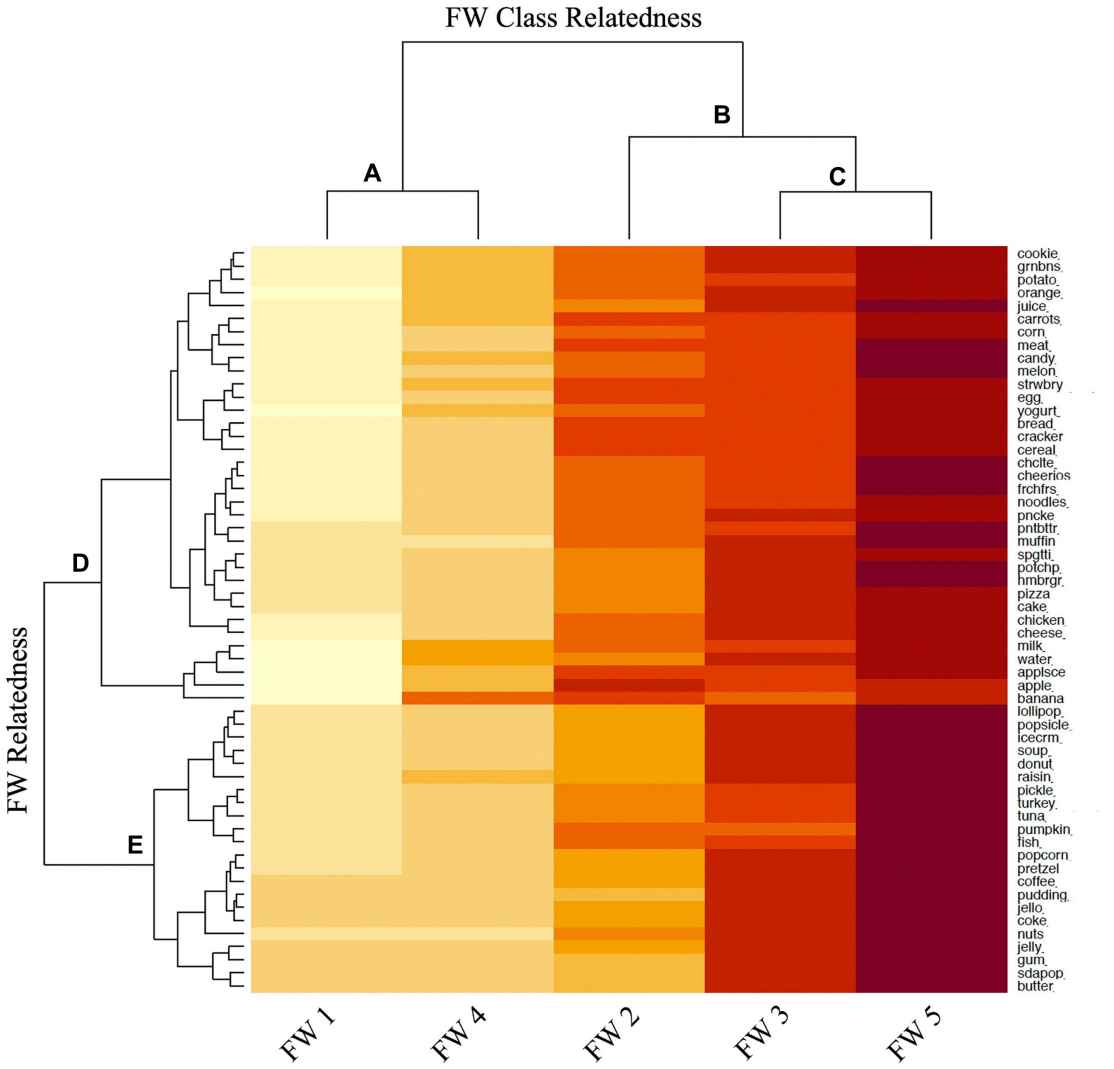
Latent class analysis of food words (FW) heat map. In order of increasing understanding of food words: FW Class 1 = low understanding of adequacy and moderation food words; FW Class 4 = low-to-moderate understanding of adequacy and moderation food words; FW Class 2 = moderate understanding of adequacy and moderation food words; FW Class 3 = moderate-to-high understanding of adequacy and moderation food words; FW Class 5 = high understanding of adequacy and moderation food words. The dendrogram displays the similarity or relatedness of the derived classes **(A–C)** and the similarity of individual food words understood in each class **(D,E)**.

Figures 2, 3 display the heat maps of the CFO and FW best fit models. The dendrogram of the LCA results are also presented. The dendrogram displays the similarity or relatedness of the derived classes (Figures 2A–C) and the similarity of individual complementary food items or words understood in each class (Figures 2D,E). For example, in Figure 2A, CFO 3 and CFO 4 are most closely related to each other, whereas CFO classes 1, 5 and 2 are most closely related (Figures 2B,C). Furthermore, complementary food items clustering together in the upper portion of the heat map, denoted by Figure 2D, are predominantly adequacy food items (e.g., cereal, chicken, and apple), whereas those clustered together in the lower portion, denoted by Figure 2E, are predominantly moderation food items (e.g., ice cream, candy, and soda pop). A similar pattern of food words understood by the index child are shown in Figure 3, where words clustered together in Figure 3D are mostly food words representing adequacy foods and those in Figure 3E are mostly food words representing moderation foods.

Each best fit model was reviewed, and derived classes were described by the degree of membership of each food item or word in each class. Degree of membership is indicated by the intensity of color shading in the heat maps (low, low-to-moderate, moderate, moderate-to-high, and high) and whether the predominant food items or words represented adequacy or moderation foods. By this classification scheme, the following labels were assigned to the derived CFO classes, in order of increasing complementary food exposure: CFO Class 3 = low exposure to adequacy and moderation complementary foods; CFO Class 4 = low-to-moderate exposure to adequacy and moderation complementary foods; Class 5: moderate exposure to adequacy and moderation complementary foods; CFO Class 2 = moderate-to-high exposure to adequacy and moderation complementary foods; CFO Class 1 = high exposure to adequacy and moderation complementary foods. The following labels were assigned to the derived FW classes, in order of increasing understanding of food words: FW Class 1 = low understanding of adequacy and moderation food words; FW Class 4 = low-to-moderate understanding of adequacy and moderation food words; FW Class 2 = moderate understanding of adequacy and moderation food words; FW Class 3 = moderate-to-high understanding of adequacy and moderation food words; FW Class 5 = high understanding of adequacy and moderation food words.

## Results

Among the 344 caregiver-child dyads included in the analytic sample, 150 children were of ages less than 12 months, 100 were between 12 months to 18 months, and 94 were of ages 18 to 26 months. The analytic sample included 201 male children (58.4%) and 143 female children (41.6%). For descriptive purposes, caregiver respondent and index child sociodemographic characteristics are presented in Table 1, organized by predicted FW classes. Total foods offered and total words understood are also presented for the total sample and by predicted FW class. Specifically, age of the index child and household income significantly differed across FW classes, such that older infants (>12 months) and toddlers (>18 months) and children living in higher earning households (>$50,000) were more likely to belong to the moderate-to-high and high understanding of FW classes. By nature of the clustering analysis, mean number of total FW understood was significantly different by FW class. The mean number of complementary foods offered also differed significantly by FW class, where, on average, a higher number of foods was offered to children in the moderate-to-high and high understanding of FW classes.

**Table 1:** Descriptive information for index child-caregiver dyads by derived class of understanding of food words.

	Total	Understanding of Food Words^1^					*p*-value^2^
		Low (*n* = 102)	Low-to-moderate (*n* = 48)	Moderate (*n* = 98)	Moderate-to-high (*n* = 68)	High (*n* = 28)	
Index child							
Age group, *n* (%):							
<12 months	150 (43.6)	69 (67.6)	21 (43.7)	29 (29.6)	28 (41.2)	3 (10.7)	<0.001
12–18 months	100 (29.1)	24 (23.5)	16 (33.3)	32 (32.6)	18 (26.5)	10 (35.7)	
>18 months	94 (27.3)	9 (8.8)	11 (23.0)	37 (37.8)	22 (32.3)	15 (53.6)	
Sex (female), *n* (%)	143 (41.6)	42 (41.2)	20 (41.7)	40 (40.8)	29 (42.6)	12 (42.9)	0.99
Birthweight (lbs), mean (*SD*)	7.7 (2.6)	7.3 (1.6)	7.6 (1.9)	8.1 (3.0)	7.8 (3.5)	7.7 (2.0)	0.35
Ever breastfed, *n* (%)							
Yes	283 (82.3)	83 (81.4)	40 (83.3)	77 (78.6)	63 (92.6)	20 (71.4)	0.08
No	61 (17.7)	19 (18.6)	8 (16.7)	21 (21.4)	5 (7.4)	8 (28.6)	
Caregiver respondent							
Age (years), mean (*SD*)	31.5 (6.3)	31.0 (6.2)	32.0 (7.6)	31.6 (5.7)	31.8 (6.7)	31.8 (5.6)	0.85
Sex (female), *n* (%)	242 (70.3)	78 (76.5)	31 (64.6)	71 (72.4)	38 (55.9)	24 (85.7)	0.05
Highest educational attainment, *n* (%):							
High school degree or less	90 (26.2)	29 (28.4)	14 (29.2)	24 (24.5)	18 (26.5)	5 (17.9)	0.80
More than high school degree	254 (73.8)	73 (71.6)	34 (70.8)	74 (75.5)	50 (73.5)	23 (82.1)	
Race and ethnicity, *n* (%):							
Non-Hispanic White	251 (73.0)	80 (70.4)	38 (79.2)	64 (65.3)	48 (70.6)	21 (75.0)	0.32
Non-Hispanic Black	59 (17.1)	12 (11.8)	4 (8.3)	24 (24.5)	17 (25.0)	2 (7.1)	
Hispanic/other/more than one race	34 (9.9)	10 (17.8)	6 (12.5)	10 (10.2)	3 (4.4)	5 (17.9)	
Household income, *n* (%):							
<$26,500	64 (18.7)	18 (17.7)	9 (18.7)	24 (24.5)	9 (13.4)	4 (1.2)	0.02
$26,500–$50,000	84 (24.5)	28 (27.4)	10 (20.8)	28 (28.6)	10 (14.9)	8 (28.6)	
$50,000–$100,000	102 (29.7)	36 (35.3)	11 (23.0)	26 (26.5)	18 (26.9)	11 (39.3)	
>$100,000	93 (27.1)	20 (19.6)	18 (37.5)	20 (20.4)	30 (44.8)	5 (17.9)	
# of children in home, mean (*SD*)	1.0 (1.1)	0.9 (1.1)	1.0 (1.0)	1.1 (1.2)	1.2 (1.2)	1.0 (0.8)	0.40
# of adults in home, mean (*SD*)	1.3 (0.9)	1.4 (0.9)	1.4 (0.9)	1.3 (0.9)	1.4 (1.0)	1.2 (0.9)	0.65
Food words understood, mean (*SD*)	29.9 (23.0)	0.9 (1.6)	15.9 (5.9)	56.5 (1.2)	43.0 (6.0)	34.9 (6.4)	<0.001
Compl. foods offered, mean (*SD*)	22.3 (14.7)	14.0 (11.7)	16.4 (10.7)	26.1 (15.8)	33.4 (12.6)	23.4 (9.1)	<0.001

^1^Low understanding of adequacy and moderation food words = FW Class 1; Low-to-moderate understanding of adequacy and moderation food words = FW Class 4; Moderate understanding of adequacy and moderation food words = FW Class 2; Moderate-to-high understanding of adequacy and moderation food words = FW Class 3; high understanding of adequacy and moderation food words = FW Class 5. ^2^Comparisons across Food-related Receptive Language classes performed via Cochran–Mantel–Haenszel tests for general association of categorical variables and ANOVA for mean comparisons of continuous variables across classes.

**Figure 4:**
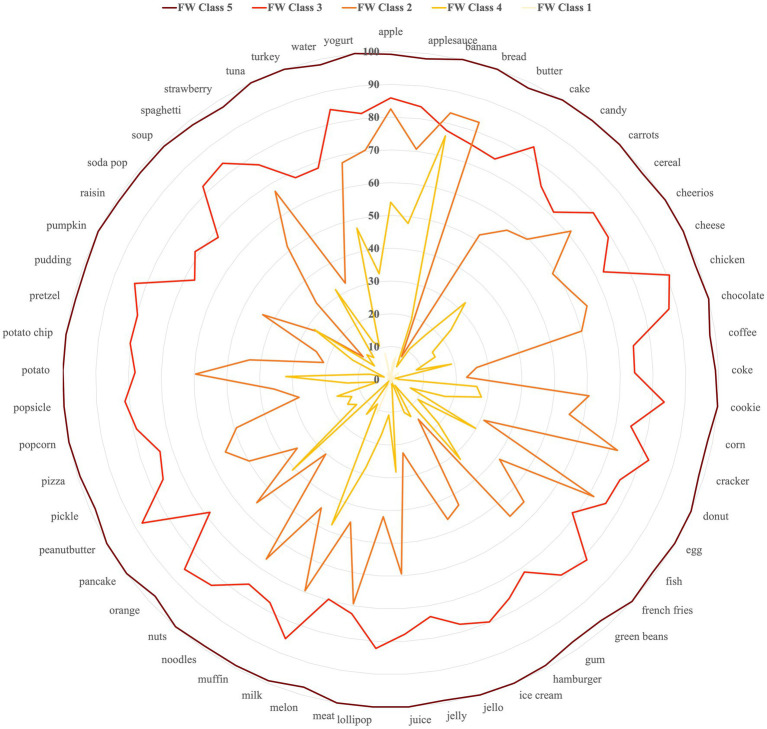
Spider plot of individual food word probabilities (0–100) by food words (FW) class membership. Color intensity of lines is matched to classes displayed in Figure 3. Food words with higher probabilities indicate that a majority of the children grouped in the given cluster understood the word per caregiver report on the modified MB-CDI.

Figure 4 provides details of the FW classes with a spider plot of the probabilities of membership for each food word in each class. Here, the group of children clustered in FW Class 5 (outer web) were perceived to understand most of the food words queried in the MB-CDI assessment (high understanding). Children in FW Class 5 were mostly older (54% ages >18 months; Table 1), which may explain the high level of food word understanding. Conversely, in the inner most web of the spider plot are the probabilities of understanding food words in FW Class 1. Children in this class were perceived to understand the least number of food words [mean (SD) 0.9 (1.6)] and, as expected, were predominantly infants (68% ages <12 months).

**Figure 5:**
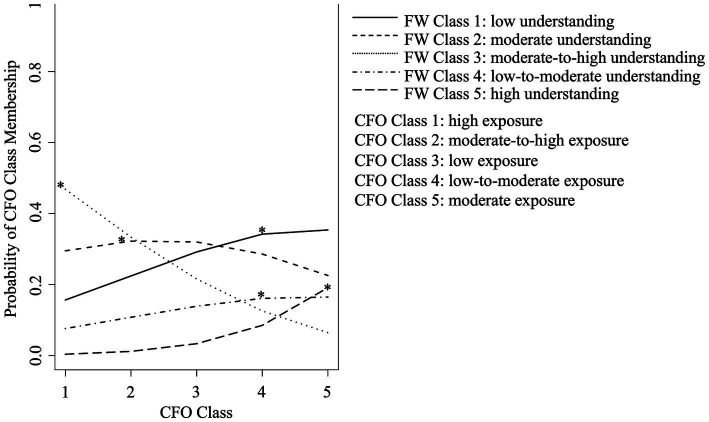
Food word (FW) class-by-complementary foods offered (CFO) class membership probabilities. FW classes denote level of understanding of food words for adequacy and moderation foods. CFO classes denote level of exposure to adequacy and moderation complementary foods. Asterisk (^*^) denotes highest probability of membership.

Figure 5 displays the probability of FW class-by-CFO class membership; or the probability of being in each FW class, given membership in each CFO class. The overall pattern of class-by-class membership suggests that infants and toddlers who had a greater understanding of food-related words were exposed to a larger number of complementary foods, both adequate and moderation foods. For example, children in FW class 3, whose understanding of food words was moderate-to-high, were most likely to be assigned to CFO class 1, with high exposure to adequacy and moderation complementary foods (probability _CFO1_ = 0.47). Children in FW class 5 with high understanding of food words were most likely to be assigned to CFO class 5, with moderate exposure to adequacy and moderation complementary foods (probability _CFO5_ = 0.19). Children in FW class 2, who were perceived to have moderate understanding of food words were most likely to be assigned to CFO class 2, with moderate-to-high exposure to adequacy and moderation complementary foods (probability _CFO2_ = 0.32). Conversely, children in FW classes 1 and 4, who had low and low-to-moderate understanding of food words were most likely to also be assigned to CFO class 4, with low-to-moderate exposure to adequacy and moderation complementary foods (probability _CFO4_ = 0.43 and 0.16, respectively).

## Discussion

In the current study, we found evidence supporting a possible relationship between food-related exposure and food-related language acquisition in infants and toddlers. Specifically, we observed that caregiver offerings of a greater variety of foods during the complementary feeding period corresponded to higher probability of the young child being perceived to understand a larger vocabulary of food-related words. Given the age range of infants and children included in the current study, our preliminary results may be capturing a facet of early life word-learning that is specific to food-related vocabulary.

Word-learning is the process of assigning meaning to words and word forms and is a significant driver of vocabulary size and development over time. Multiple speech-related mechanisms are thought to interact to promote word-learning (20), but evidence from several in-depth studies of early word-learning indicates that early vocabularies, especially that which develops in infancy, are primarily learned from repetitious exposure to isolated words (21–23). Although isolated words are shown to be used infrequently in infant and child-directed speech (24–26) overall, the frequency of isolated word use may vary depending on the context or environment. For example, during mealtimes, an isolated word for a food item may be repeated more frequently by the caregiver, compared to other times of the day, thus, likely providing frequent exposure to the respective food word, in the eating context.

In addition to frequency and repetition as central aspects of early word learning (27, 28), use of referential cues by caregivers (e.g., showing attention or gaze to an object) coupled with speaking the referent isolated word for the object also influences word-learning (29). Again, within the context of mealtimes, the act of feeding or offering food likely exposes infants and children to frequent food word pairings to caregiver referential cues or prompts to the food item in question. Indeed, a recent study by Barrett et al. revealed that caregivers use numerous supportive and engaging verbal prompts while feeding their infants and toddlers with high interindividual variability, i.e., some caregivers were completely silent during feeding whereas others spoke continuously (30). However, it is important to emphasize that no study to date has specifically examined the frequency of isolated food words in naturalistic infant and child-directed speech or quantified the use and frequency of referential cues to food items during mealtimes. Therefore, the specific mechanism underlying food-related word-learning in infants and toddlers in the current study remains unknown. Future research should investigate food-related single-word utterances during mealtimes when caregivers practice naturalistic infant-directed speech so to quantify the magnitude of isolated word exposure that infants and toddlers experience.

Other factors not accounted for in our study may also contribute to food-related language development. For example, a recent study by Webber et al. (31) found that children of caregivers who offered foods from the family table during mealtime had greater language acquisition, suggesting yet another form of interaction that may drive language development. Although, more research is needed to expand our understanding of contributors to food-related language development, our current results, like those of Webber et al. (31), align with the interactionist framework of language, whereby greater frequency of introduction to diverse, complementary foods (the repeated and reinforced interaction) is related to understanding of a larger number of food words (the development of food-related language). Together with our prior work showing that greater understanding of food words was related to greater acceptance of a novel food, independent of repeated exposure to the novel food itself (11), and from the observations made in the current study, we hypothesize that the variety of complementary foods fed contributes, in part, to greater acquisition of food-related language, which then promotes, in part, eating behaviors. Again, however, this pathway has not been directly tested and future research should investigate this possible feedforward process in early development of eating behaviors via feeding and language using direct observation and experimental designs.

### Strengths

The primary strength of our study is that it provides novel, preliminary evidence of the potential influence of complementary feeding on language development. A further strength of our study is its generalizability to caregivers of young children in the United States, as it included a large sample size of socioeconomically and geographically diverse respondents. Additionally, while adapted to focus on food-related language only, our survey’s language module was derived from a validated clinical screening measure (MB-CDI) specifically applied to children in the age range assessed. Finally, while the included food words were limited, the diversity of foods they represented was large including both adequacy and moderation items.

### Limitations

Within the context of the novelty and strengths of our study, several limitations must be considered when interpreting our results. First, both complementary feeding and language development are highly dependent on age of the child, where older children are likely offered a greater variety of complementary foods and are also developmentally accelerating their language learning. In the current study we did not normalize language to age due to the modified nature of our food words language assessment for which age-standardized norms are not available. Thus, we expected that the children with the largest food vocabulary would be children in the oldest age category (>18 months; Table 1). However, despite this, we did observe greater variability in age across the classes of food word understanding than expected. This may be due, in part, to significant variability in complementary feeding practices in the United States, specifically in age at introduction of complementary foods as well as the variability to which caregivers converse with young children during feeding. Several recent reports have shown that a third to half of infants are introduced to complementary foods before 6 months of age (32, 33). Based on the interactionist framework of language, this variability in introduction and complementary food exposure could propel development of food word lexicons at younger ages. Indeed, we observed that the group of children clustered in FW Class 3, having moderate-to-high understanding of food words, were predominantly younger infants (41% ages <12 months; Table 1). However, due to the caregiver-report of language abilities, this could also be due to caregiver misperception of children’s understanding of language.

Second, while the MB-CDI is a standardized and well-adopted language screening assessment, its validity has been called in to question recently due to its basis in caregiver-report (34). Additionally, due to its purpose as a broad language screener, the MB-CDI does not include food words that necessarily reflect the current complementary feeding landscape or what may be considered age-appropriate foods that align with current feeding recommendations. Consequently, because we matched the foods listed on the modified FFQ to our modified MB-CDI, the modified FFQ also does not capture the full breadth of foods used in complementary feeding practices. Moreover, we examined only vocabulary, specifically nouns. Thus, we cannot interpret our findings within the context of other elements of language like morphology or syntax. Therefore, our results are likely an underestimation of food word understanding within the age groups examined.

Finally, due to our modification of the MB-CDI to focus specifically on food words, the resulting assessment was a crude language measure and was not a comprehensive examination of language skills with more objective observed measures of children’s understanding. However, this was by design and our results should be interpreted within the hypothesis generating scope of our analysis. Furthermore, although we excluded caregiver-child dyads where the child was reported to have a developmental disability or was born prematurely (<37 weeks’ gestation), both exposure to complementary feeding and language acquisition could be affected by neurodevelopmental processes not measured in the current study.

## Conclusion

In this hypothesis-generating study, we showed that greater exposure to a complementary food corresponded to greater food word understanding in infants and toddlers. These results represent a preliminary step in examining the role of early life feeding experiences in language development. Upon confirmation of our findings from other independent cohorts and expansion to test the full pathway from complementary feeding, food language understanding, and eating behaviors, this evidence may help to design novel feeding and eating interventions that utilize food-related language development as a method by which to familiarize young children to foods and reduce potential fear associated with food novelty.

## Data availability statement

The datasets presented in this article are not readily available because data may be requested from the authors and shared in accordance with the restrictions specified by participants in the consent forms. Requests to access the datasets should be directed to SJ, susan.johnson@cuanschutz.edu.

## Ethics statement

The studies involving humans were approved by Colorado Multiple Institutional Review Board. The studies were conducted in accordance with the local legislation and institutional requirements. The participants provided their written informed consent to participate in this study.

## Author contributions

AS, ML, and SJ designed and conducted the study and conceived of the idea for the current manuscript. AS conducted the statistical analysis and drafted the full manuscript. ML, RC-S, and SJ reviewed and contributed significantly to revisions of the draft manuscript. All authors contributed to the article and approved the submitted version.
