# Supplementary material for: Complementary food exposure and children’s early understanding of food words: the approaching eating through language (APPEAL) study
Source: Front Nutr. 2024 May 28;11:1237698. doi: 10.3389/fnut.2024.1237698 (PMC11165136; doi:10.3389/fnut.2024.1237698)
Supplement: Supplementary file 1 [file Table_1.DOCX]

Supplementary Table 1. Food words included in modified MacArthur Bates language assessment and Food Frequency Questionnaire.

| apple^1^ | lollipop |
| --- | --- |
| applesauce^1^ | meat^1^ |
| banana^1^ | melon^1^ |
| bread | milk^1^ |
| butter | muffin^1^ |
| cake^1^ | noodles^1^ |
| candy^1^ | nuts^1^ |
| carrots^1^ | orange^1^ |
| cereal^1^ | pickle |
| chocolate^1^ | pizza |
| cheerios^1^ | pancake^1^ |
| cheese^1^ | peanut butter^1^ |
| chicken^1^ | popcorn |
| coffee | popsicle^1^ |
| coke^1^ | potato^1^ |
| cookie^1^ | potato chip |
| corn^1^ | pretzel^1^ |
| cracker^1^ | pudding^1^ |
| donut^1^ | pumpkin^1^ |
| egg^1^ | raisin^1^ |
| fish | soda pop^1^ |
| french fries^1^ | soup^1^ |
| green beans^1^ | spaghetti^1^ |
| gum | strawberry^1^ |
| hamburger^1^ | tuna |
| ice cream^1^ | turkey^1^ |
| jello | water |
| jelly | yogurt^1^ |
| juice^1^ |  |

1: Food words overlap with most common foods offered to infants and toddlers and reported by the FITS study ^15,16^.
